# QARIP: a web server for quantitative proteomic analysis of regulated intramembrane proteolysis

**DOI:** 10.1093/nar/gkt436

**Published:** 2013-05-31

**Authors:** Dmitry N. Ivankov, Natalya S. Bogatyreva, Peter Hönigschmid, Bastian Dislich, Sebastian Hogl, Peer-Hendrik Kuhn, Dmitrij Frishman, Stefan F. Lichtenthaler

**Affiliations:** ^1^Department of Genome Oriented Bioinformatics, Technische Universität München, Wissenschaftszentrum Weihenstephan, Maximus-von-Imhof Forum 3, 85354 Freising, Germany, ^2^Laboratory of Protein Physics, Institute of Protein Research, Russian Academy of Sciences, Institutskaya Str. 4, 142290 Pushchino, Moscow Region, Russia, ^3^German Centre for Neurodegenerative Diseases (DZNE), Max Lebsche Platz 30, 81377 Munich, Germany, ^4^Neuroproteomics, Klinikum rechts der Isar, Technische Universität München, Max-Lebsche-Platz 30, 81377 Munich, Germany, ^5^Helmholtz Center Munich—German Research Center for Environmental Health (GmbH), Institute of Bioinformatics and Systems Biology, Ingolstädter Landstraße 1, D-85764 Neuherberg, Germany and ^6^Munich Cluster for Systems Neurology (SyNergy), Adolf-Butenandt-Institut, Ludwig-Maximilians-Universität München, Schillerstrasse 44, 80336 Munich, Germany

## Abstract

Regulated intramembrane proteolysis (RIP) is a critical mechanism for intercellular communication and regulates the function of membrane proteins through sequential proteolysis. RIP typically starts with ectodomain shedding of membrane proteins by extracellular membrane-bound proteases followed by intramembrane proteolysis of the resulting membrane-tethered fragment. However, for the majority of RIP proteases the corresponding substrates and thus, their functions, remain unknown. Proteome-wide identification of RIP protease substrates is possible by mass spectrometry-based quantitative comparison of RIP substrates or their cleavage products between different biological states. However, this requires quantification of peptides from only the ectodomain or cytoplasmic domain. Current analysis software does not allow matching peptides to either domain. Here we present the QARIP (Quantitative Analysis of Regulated Intramembrane Proteolysis) web server which matches identified peptides to the protein transmembrane topology. QARIP allows determination of quantitative ratios separately for the topological domains (cytoplasmic, ectodomain) of a given protein and is thus a powerful tool for quality control, improvement of quantitative ratios and identification of novel substrates in proteomic RIP datasets. To our knowledge, the QARIP web server is the first tool directly addressing the phenomenon of RIP. The web server is available at http://webclu.bio.wzw.tum.de/qarip/. This website is free and open to all users and there is no login requirement.

## INTRODUCTION

Regulated intramembrane proteolysis (RIP) is a basic cellular mechanism controlling the communication between cells and their environment (1). RIP is a two-step process that involves the sequential cleavage of membrane proteins by ectodomain shedding and intramembrane proteolysis. In the first step of RIP, specific membrane-bound proteases (also known as sheddases), release the ectodomain into the extracellular space. In the second step of RIP, the remaining membrane-bound fragment is then cleaved within the membrane by members of the intramembrane protease families, which releases the intracellular domain into the cytosol (2). The liberated protein fragments may then serve as para- and endocrine signaling molecules in the extracellular space and as transcription factors upon release into the cytosol. The interplay of RIP proteases and their substrates thus contributes to various aspects of cell–cell communication. In addition, deregulation of the RIP process is found under many pathophysiological conditions. The proteases and their corresponding substrates therefore serve as putative biomarkers and as target molecules for pharmacologic interference. RIP is mediated by over 30 different proteases, such as members of the ADAM family of proteases, BACE1 and GXGD type proteases (3–5). It affects more than 1000 membrane proteins, but for the majority of substrates the responsible protease is unknown.

Recent advances in mass-spectrometry (MS) based proteomics have extended its applicability to diverse areas of biological research. Beyond identification of proteins present in a cell and their modifications (6), MS has been successfully applied to discover novel genes (7), correct gene starts (8), detect non-ribosomally synthesized peptides (9), as well as to identify signal (10,11) and transit (12) peptides. The current accuracy of mass spectrometry makes it suitable for studying protein expression under different conditions (13). Modern quantitative proteomics allows for the investigation of almost complete proteomes within a reasonable timeframe, and is thus increasingly exploited for the analysis of RIP. However, in order to identify novel RIP substrates and to accurately monitor the RIP process on a membrane proteome-wide scale, differential quantitation of RIP products within the chosen compartment is needed. For example, when comparing a lysate of a control cell line with a sheddase knockout cell line, peptide ratios for the intracellular domain of the protease substrate may remain the same, but the ratios of peptides mapping to the extracellular region will be dramatically different. The differential analysis therefore results in a more specific and sensitive quantitation, especially if using data that is oversampled, i.e. where the peptide sequence of most proteins is covered to a larger extent.

Here we present a descriptive web server, QARIP (Quantitative Analysis of Regulated Intramembrane Proteolysis) that specifically aims at analysing the RIP-induced perturbations in the cell by means of MS-based peptide identification. QARIP features a simple and straightforward workflow applied to user-supplied data in order to aid in the evaluation of RIP data, by automatically assigning detected peptides to extracellular, intracellular or transmembrane domains. Peptides that are assigned to the extracellular domain allow the monitoring of ectodomain shedding events, whereas peptides assigned to the transmembrane domain and intracellular part are used to analyse the intramembrane proteolysis. When studying secretomes, a quick scan of the dataset for the presence of extracellular and the absence of intracellular peptides serves as a quality control for the purity of the sample, ruling out cytosolic contaminants, e.g., through apoptosis or detached cells. QARIP supports the direct upload of data in tab-delimited text format. It integrates Gene Ontology (GO) information as well as other useful filter options. This allows a quick categorization of the dataset into type I, type II and multi-pass transmembrane proteins as well as into glycosylated and non-glycosylated proteins, thus providing a quick separation of the dataset into classically secreted and non-classically secreted proteins as well as the proteins undergoing RIP. Annotation of protein topology is based on already existing data and is combined with additional information provided by topology prediction algorithms. Recent breakthroughs in targeted and non-targeted proteomics as well as in biochemical techniques enriching for secreted proteins allow the quantitative analysis of hundreds to thousands of proteins within a single experimental setup. Data analysis is facilitated by various software applications, however none of them are targeted to the specific nature of RIP, and the manual inspection of individual peptides is cumbersome due to the large amount of data. QARIP is the first freely available software that is targeted toward the needs of the rapidly growing community of MS-based analysis of ectodomain shedding and intramembrane proteolysis.

## METHOD OVERVIEW

### Input data

The QARIP input must contain quantitative information about peptides detected by tandem mass spectrometry under two different conditions, with one condition of interest (e.g. when a specific sheddase is knocked out) and the other condition being a control one. The server returns the list of proteins with the levels of enrichment/depletion of detected peptides in the cytoplasmic and extracellular domains. The tab separated input file must contain the identifier of the protein harboring the peptide identified by MS, the peptide sequence and MS/MS peak intensity corresponding to the peptide under two different conditions in the first four columns. The first header line of the input file as well as any additional columns are ignored.

### QARIP workflow

Once the input data have been uploaded the user receives a unique eight-symbol job identifier that can be later used to access the results of calculations. Additionally, the user is notified by e-mail containing the active link. The data are deleted from the web server after one week since the last activity.

The server can accept different types of user-provided protein identifiers (UniProt accession code or protein identifier (14), NCBI Id (http://www.ncbi.nlm.nih.gov/), IPI Id (http://www.ebi.ac.uk/IPI/UnProtFormat.html)). The latter two are mapped to UniProt ones using mapping file from the stand-alone UniProt package. The protein sequence as well as the associated gene names, the transmembrane topology, and GO terms are then extracted from UniProt. For all proteins, QARIP makes a sequence-based prediction using the stand-alone version of the Phobius algorithm (15). The transmembrane annotation from UniProt is simplified as follows: signal, transit and propeptides are merged into one ‘signal’ category, while all non-cytoplasmic types of annotation (i.e. extracellular, lumenal, periplasmic, etc.) are merged into an ‘extracellular’ category.

Peptides detected in MS/MS experiment are then mapped onto the protein sequence. From all protein isoforms one isoform covering all detected peptides is selected as representative (the longest one if several isoforms cover all found peptides). If the detected peptides cannot be attributed to a single isoform, the minimal number of isoforms covering all detected peptides is chosen. For each peptide the ratio of intensities between two conditions is calculated and then for each isoform these ratios are averaged over all detected peptides, separately for the extracellular and cytoplasmic portions of the protein.

### Presentation layout

**Figure 1.**
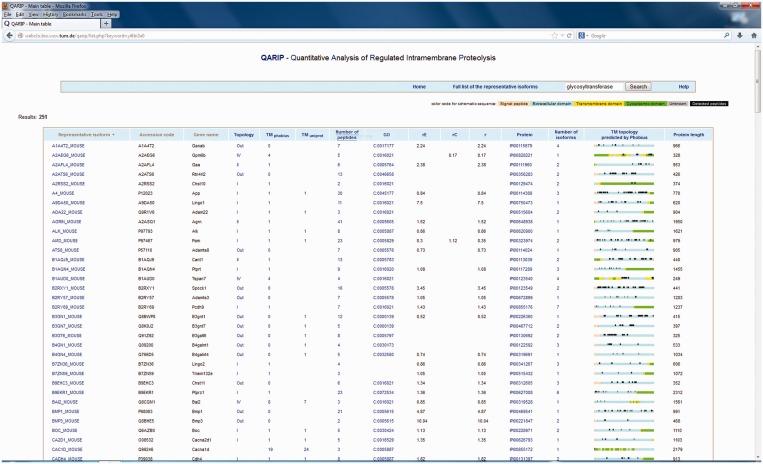
The list of representative isoforms shown for an example experiment on neuronal secretome (16). The page lists the UniProt identifier of the representative isoform, UniProt accession code, gene name, topology, number of transmembrane helices (‘TM’) predicted by Phobius and annotated in UniProt, number of detected peptides, GO terms, average ratio of peptide intensity both for extracellular and cytoplasmic parts as well as for the whole protein (rE, rC and r, respectively), user-provided protein identifier, overall number of isoforms for the particular protein and the illustration of protein transmembrane topology predicted by Phobius (15) with detected peptides highlighted.

The table of representative isoforms (Figure 1) allows to identify proteins having detected peptides both in extracellular and cytoplasmic parts (A2AEG6_MOUSE in the Figure 1). The comparison of the average enrichment ratio upon knockout for extracellular and cytoplasmic parts (rE and rC, respectively) indicates whether the protein is a substrate of the knocked out sheddase or not.

**Figure 2.**
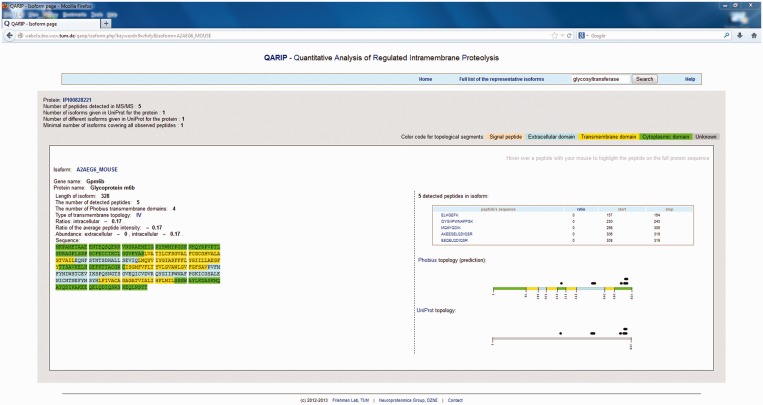
Individual isoform page. The page provides basic information about the isoform, its sequence with colored transmembrane domains (topology predicted by Phobius), table of detected peptides and graphical illustration of the transmembrane topology according to Phobius and UniProt, with detected peptides schematically represented as black boxes. Detected peptides, both given in the table and depicted in the pictures, can be hovered over to highlight the position of the peptide in the protein sequence.

Individual isoform page (Figure 2) gives further information about the contribution of each peptide to the intensity ratio. The individual protein page is designed in the same way as the isoform page does, but includes information about all isoforms available for the particular protein, with the representative isoform going first.

### Sample preparation and mass spectrometry

HEK293E cells stably expressing BACE1-HA or the empty control vector were grown in heavy (13C6, 15N2-labeled lysine) or light SILAC media (SILANTES), respectively, for at least six passages. The conditioned media were collected, pooled, cleared by centrifugation and concentrated using ultrafiltration columns (Amicon) with a 3 kDa nominal molecular weight cut-off to a final volume of 100 µl. The concentrated medium was mixed with 100 µl of 2× SDT buffer (2% SDS, 100 mM DTT, 100 mM Tris pH 7.6) and incubated at 95°C for 5 min. The conditioned media was then processed by filter-aided sample preparation as described before (17). Lys-C (Promega) was used as digestion enzyme on ultrafiltration columns with a 3 kDa nominal molecular weight cut-off. The peptides were further fractionated using Stage-Tip-based SAX fractionation into six fractions (18). LC-MS/MS analysis was carried out on a Proxeon Easy nLCII (Thermo Fisher Scientific) coupled to an LTQ Orbitrap Velos mass spectrometer (Thermo Fisher Scientific) using 4 h linear gradients. 15 cm emitter columns (New Objective) packed with C18-AQ 2,4 Nm resin (Dr Maisch GmbH) were used.

### Protein identification, quantitation and submission to QARIP

Raw files were analysed using the freely available MaxQuant Software, using the default settings (19) (Version 1.3.0.5, available at www.maxquant.org). Both biological replicates were analysed together. MaxQuant output files are in tab-delimited text format and therefore easily accessible for further processing by Microsoft Excel or other spreadsheet analysis software. The peptides.txt table generated by MaxQuant contains all the necessary information for the analysis by QARIP. Other mass spec software also provides the processed data in a text format and can be dealt with in the same manner. In our case, we imported the peptides.txt table into Microsoft Excel, filtered for peptides where light and heavy partners were identified in both biological replicates and that did not match to a database consisting of common protein contaminants (such as keratin). Peptide sequences, the normalized heavy to light ratios and the corresponding UniProt IDs were then assembled into four columns (1: UniProt ID, 2: sequence, 3: digit ‘1’, 4: heavy to light ratio), saved as a .txt file and submitted to the QARIP web server. For SILAC data, the third of the four columns should only be filled with the digit ‘1’, as the fourth column already contains the information for the ratio. In the case of a label free dataset, the measured intensities must be placed in the third and fourth column, respectively, as the server divides the values of the fourth by the third column to obtain the desired ratios.

## TYPICAL USE OF THE SERVER

The membrane-bound aspartyl protease BACE1 (*β*-site APP cleaving enzyme 1) is one of many proteases involved in the ectodomain shedding of transmembrane proteins. Ectodomain shedding releases soluble ectodomains into the extracellular space, where they act as signaling molecules or are rapidly degraded (1). Multiple BACE1 substrates have been described in the past, among them the amyloid precursor protein (APP), which is processed in a stepwise fashion by BACE1 and γ-secretase (4). The proteolytic processing of APP releases the neurotoxic Aβ peptide into the extracellular space, where it accumulates and initiates a neurotoxic cascade resulting in Alzheimer’s disease (20).

To demonstrate the usability of QARIP, we sought to identify previously known or novel BACE1 substrates in the conditioned media of HEK293 cells overexpressing either BACE1 or an empty control vector. In order to accurately quantify the expected changes, cells were grown in heavy or light SILAC media, respectively. The conditioned media were then analysed by LC-MS/MS. The heavy/light ratio for the identified peptides was subsequently submitted to QARIP, along with the corresponding peptide sequences and UniProt IDs. The ectodomains of BACE1 substrates were expected to accumulate in the conditioned media of cells overexpressing BACE1, resulting in a heavy/light ratio above 1.

**Figure 3.**
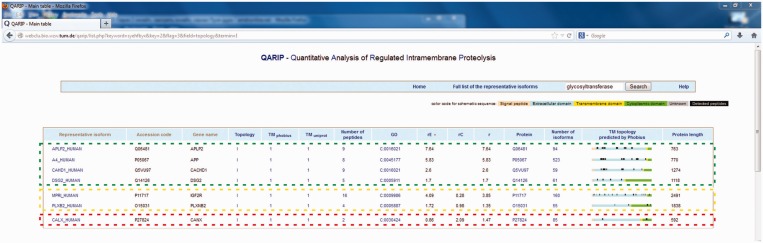
Example of QARIP use. Identification of novel BACE1 substrates (for details see the text).

Figure 3 shows several candidates identified in the case study that were enriched at least 1.35-fold (overall ratio) in BACE1 overexpressing cells. Proteins in the green box, including the known BACE1 substrates APP and amyloid like protein 2 (APLP2) (4), were only detected by peptides originating from the extracellular domain. This is the ideal case, where the overall ratio equals the extracellular ratio and reflects the differences in ectodomain shedding by BACE1, as no contaminating cytosolic peptides were detected. For the proteins shown in the yellow box, peptides from the extracellular as well as the transmembrane domain were detected. In the case of Plexin-B2 (PLXNB2), contaminating cytosolic peptides (attributable to exocytosis, apoptosis or detached cells in the conditioned media) were equally present in both conditions and decreased the overall ratio in comparison to the ratio of extracellular peptides. A similar situation was observed for the cation-independent mannose-6-phosphate receptor (IGF2R). In this case, however, there was an unequal contamination of cytosolic peptides in between the conditions, resulting in even more drastic differences between the overall and extracellular ratio. In conclusion, both proteins are putative novel BACE1 substrates, one of them having been previously identified in a similar study (21). For those proteins, the amount of ectodomain shedding is more accurately quantified using QARIP in comparison to the established workflows, where all detected peptides are used for quantification. In the case of Calnexin (CANX), encircled by the red box, the overall ratio also suggests that this protein could represent a novel BACE1 substrate. However, analysis by QARIP reveals that the ratio of extracellular peptides is not changed significantly. To conclude, the automatic annotation of peptides to the individual domains of transmembrane proteins, along with the visual presentation and filter options of QARIP, allow a quick identification of putative substrates, their accurate quantification and reveal quantitative changes not attributable to ectodomain shedding.

## FUNDING

DFG International Training and Research Group RECESS (Regulation and Evolution of Cellular Systems); Program “Molecular and Cellular Biology” of the Russian Academy of Sciences [01201358029]; DFG within the framework of the Munich Cluster for Systems Neurology [EXC 1010 SyNergy]; the BMBF project KNDD; Carl von Linde Junior Fellowship [TUM-IAS]. Funding for open access charge: DFG, Munich Cluster for Systems Neurology [EXC 1010 SyNergy].

*Conflict of interest statement*. None declared.
